# Drivers of realized satellite tracking duration in marine turtles

**DOI:** 10.1186/s40462-020-00237-3

**Published:** 2021-01-05

**Authors:** Kristen M. Hart, Jacquelyn C. Guzy, Brian J. Smith

**Affiliations:** 1 U.S. Geological Survey, Wetland and Aquatic Research Center, 3321 College Avenue, Davie, FL 33314 USA; 2 Department of Wildland Resources, Utah State University, Logan, UT 84322 USA

**Keywords:** Biologging, Telemetry, Platform terminal transmitter

## Abstract

**Background:**

Satellite tags have revolutionized our understanding of marine animal movements. However, tags may stop transmitting for many reasons and little research has rigorously examined tag failure. Using a long-term, large-scale, multi-species dataset, we evaluated factors influencing tracking duration of satellite tags to inform study design for future tracking studies.

**Methods:**

We leveraged data on battery status transmitted with location data, recapture events, and number of transmission days to probabilistically quantify multiple potential causes of failure (i.e., battery failure, premature detachment, and tag damage/fouling). We used a combination of logistic regressions and an ordinary linear model including several predictor variables (i.e., tag type, battery life, species, sex, size, and foraging region).

**Results:**

We examined subsets of data from 360 satellite tags encompassing 86,889 tracking days deployed on four species of marine turtles throughout the Gulf of Mexico, Caribbean, and Bahamas from 2008 to 2019. Only 4.1% of batteries died before failure due to other causes. We observed species-specific variation in how long tags remain attached: hawksbills retained 50% of their tags for 1649 days (95% CI 995–1800), loggerheads for 584 days (95% CI 400–690), and green turtles for 294 days (95% CI 198–450). Estimated tracking duration varied by foraging region (Caribbean: 385 days; Bahamas: 356; southern Gulf of Mexico [SGOM]: 276, northern Gulf of Mexico [NGOM]: 177). Additionally, we documented species-specific variation in estimated tracking duration among foraging regions. Based on sensor data, within the Gulf of Mexico, across species, we estimated that 50% of tags began to foul after 83 95% CI (70–120) days.

**Conclusions:**

The main factor that limited tracking duration was tag damage (i.e., fouling and/or antenna breakage). Turtles that spent most of their time in the Gulf of Mexico had shorter tracking durations than those in the Bahamas and Caribbean, with shortest durations observed in the NGOM. Additionally, tracking duration varied by species, likely as a result of behaviors that damage tags. This information will help researchers, tag companies, permitting agencies, and funders better predict expected tracking durations, improving study designs for imperiled marine turtles. Our results highlight the heterogeneity in telemetry device longevity, and we provide a framework for researchers to evaluate telemetry devices with respect to their study objectives.

**Supplementary Information:**

The online version contains supplementary material available at 10.1186/s40462-020-00237-3.

## Introduction

The use of satellite telemetry has become a standard practice in field of marine vertebrate ecology to track movements and habitat use of animals at sea [1, 2], including marine turtles (see reviews by [3–6]). These tracking datasets provide important spatio-temporal data for understanding both nearshore and ocean-basin scale movements of individuals in the marine environment [7]. Current technological advances in biologging tools allow for an increase in the scope and scale of our understanding of marine animal movements (e.g., horizontal and vertical movements over time [8];). However, finer-scale tracking data does not necessarily advance understanding of animal ecology, as there are tradeoffs between tag costs, sample size, tag failure rates [9], and study length, which can span days to years. Project goals and budgets typically dictate the type of satellite tag used, with researchers weighing expected battery life against size of tags to select the smallest tag with highest expected battery life and thus reduce the burden on the animal [10]. Few guidelines exist for ‘best in practice’ but creative tests of available tags have recently emerged (e.g., drag estimates [11]).

Ultimately, many satellite tracking studies seek to inform conservation and management strategies (e.g., [12]) thus, careful examination of factors influencing instrumented animals (i.e., [10, 11, 13, 14], including drivers of tag failure, are imperative. Yet despite exponential growth in tracking studies worldwide [8], translating these data into useful conservation messages is challenging [15] as is tracking their impact on policy [16]. Beyond identifying probable causes of failure in specific studies (e.g., ocean sunfish [17]), limited research to date has rigorously examined satellite tag failure. Instead, studies typically report various tag performance metrics. For example, causes of signal loss from transmitters routinely attached to birds, marine turtles, and marine mammals has been attributed to battery failure, salt-water switch failure, antenna breakage, tag fouling by marine algae or barnacles, animal mortality or predation, and premature detachment of tags ([18]; e.g., penguins [19], migratory birds [20], sharks [21]). However, because remotely sensed data (e.g., sensor wet/dry status) and auxiliary information (e.g., battery voltage) are now regularly relayed with locations, rather than reporting performance metrics, researchers can conduct rigorous quantitative assessments to uncover when and why tags fail [18], and ascertain what conditions seem to drive the variation in those tag failure rates. Here, we use and extend the procedures previously outlined (i.e., [18]). Our aim was to synthesize data available in our own tracking projects to assess factors influencing satellite-derived transmissions and help improve the conservation value of these tracking studies.

Here, we focused on determining drivers of realized satellite tracking durations for several species of hard-shelled marine turtles tagged at both nesting beaches and in-water sites, and tracked for over 12 years in the Gulf of Mexico, Caribbean, and Bahamas. We examined four main causes influencing tracking duration: battery failure, premature detachment, tag fouling, or tag damage, and incorporated the influence of species, gender, turtle size, tag model, and resident foraging location on tracking duration. Our results inform study design for future research by providing information on realistic tracking durations for proposed projects on marine turtles, but also provide a framework for evaluating the diverse causes of tag failure that can be applied to a wide variety of terrestrial and marine taxa.

## Methods

**Fig. 1:**
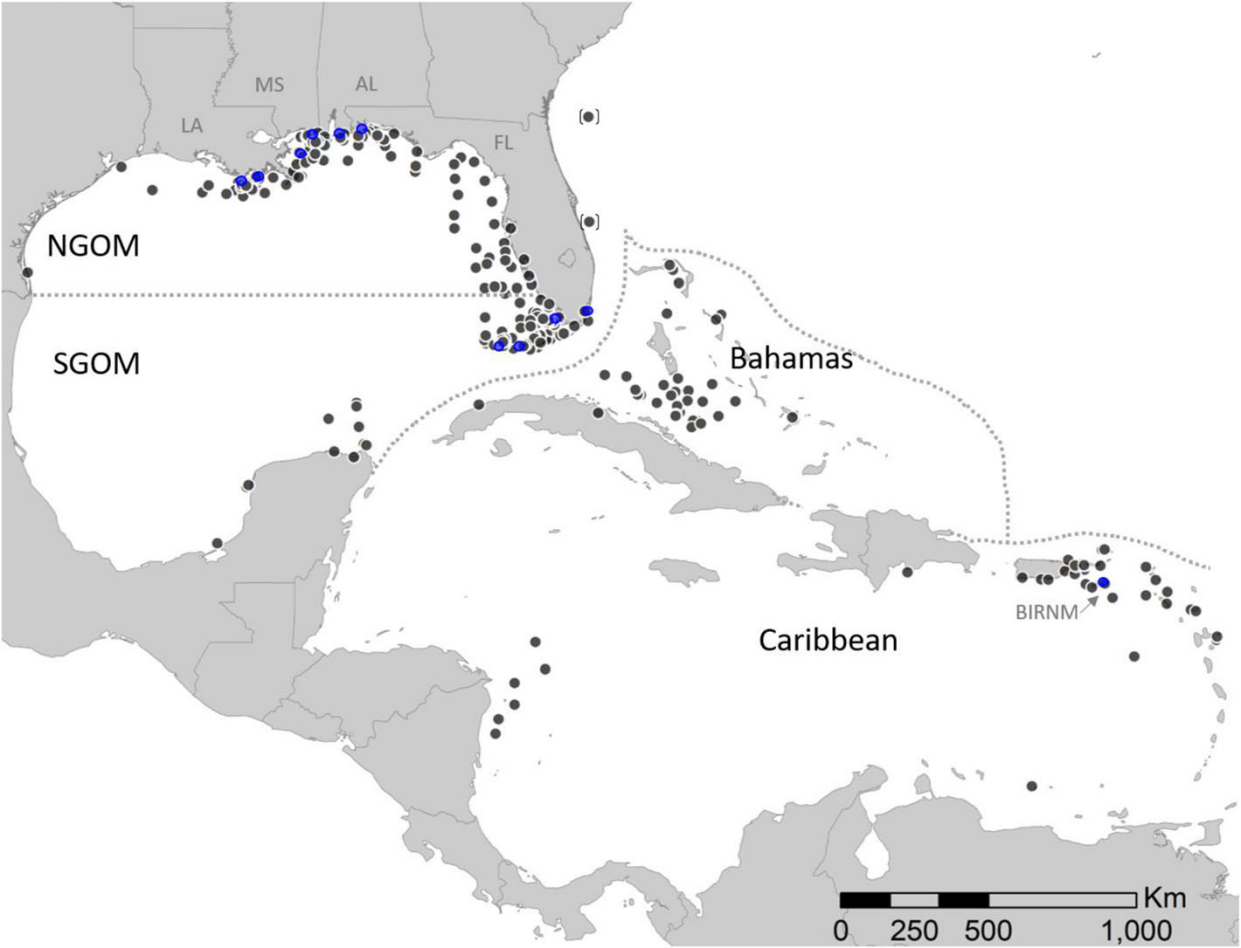
Map indicating location of marine turtles (black dots, *n* = 333) within designated foraging areas in the northern Gulf of Mexico (*n* = 113 turtles), southern Gulf of Mexico (*n* = 134), Bahamas (*n* = 40), and Caribbean (*n* = 46). For clarity species are not depicted [loggerhead (*n* = 186), green turtle (*n* = 72), hawksbill (*n* = 42) and Kemp’s ridley (*n* = 33)]. Two turtles off the of east coast of Florida (black dots enclosed in brackets) forage within the Atlantic and are not included in analyses. Blue dots indicate locations where turtles have primarily been tagged: Louisiana (Ship Shoal, Port Fourchon-Belle Pass, Chandeleur Islands), Mississippi (Pascagoula), Alabama (Gulf Shores), Florida (Dry Tortugas, Biscayne and Everglades National Parks), and the U.S. Virgin Islands (Buck Island Reef National Monument-BIRNM)

We deployed satellite tags on four species of imperiled marine turtles (green turtles *Chelonia mydas,* loggerheads *Caretta caretta*, hawksbills *Eretmochelys imbricata,* and Kemp’s ridleys *Lepidochelys kempii*) captured throughout the Gulf of Mexico and Caribbean from May 2008 through July 2019 (Fig. 1). Tagging primarily occurred at several locations within the Gulf of Mexico, including Louisiana (Ship Shoal, Port Fourchon/Belle Pass, and Chandeleur Islands), Mississippi (Pascagoula), Alabama (Gulf Shores), Florida (Dry Tortugas, Biscayne, and Everglades National Parks), and the U.S. Virgin Islands (Buck Island Reef National Monument). See Hart et al. [22–25] for more details on study sites and tagging locations.

### Capture, marking, and satellite tag attachment

We captured turtles using both land-based interception of nesting females after nesting and non-nesting emergences, whereupon we restrained them with a portable corral (96.5 cm wide × 67.3 cm height). We also captured turtles using several in-water methods (i.e., hand captures via snorkeling [26], rodeo or turtle-jumping [27, 28]; trawling, and dipnet [24, 29]). Upon capture each new turtle was given a passive integrated transponder (PIT) tag in the shoulder or front flipper (Biomark, Boise, ID; models 12 mm tag = BIO12.B.01 V2 PL.SY and 8 mm tag = BIO8.B.03 V1 PL.SY) and individually numbered Inconel flipper tags (National Band and Tag, Newport, KY; model 681) following established protocols (NMFS-SEFSC 2008). For all turtles we took standard measurements including curved (CCL) and straight (SCL) carapace lengths.

Turtles were outfitted with satellite transmitters on their anterior carapace using established protocols [30] and we limited the epoxy (Superbond™) footprint to minimize drag to turtles [11]. Refer to Additional file 1 for detailed information on tag attachment protocol. Most tags were not coated with anti-fouling paint until recent years. We used various models of satellite tags from Wildlife Computers (Redmond, WA, USA; SPOT, SPLASH, and Fastloc GPS tags; Additional file 2]. We programmed tags to send location data daily for all Kemp’s ridleys and all green turtles tagged in the Caribbean. Likewise, from 2008 to 2010 we programmed tags to transmit daily for loggerheads and hawksbills, and to conserve battery life we duty-cycled these beginning in 2011. More specifically, in 2011 we programmed tags on nesting female loggerheads to transmit every 3rd day (from 1 October – 31 March) and in 2012, tags on nesting hawksbills were set to every 3rd day (from 1 December-30 April). To further conserve battery life, we also imposed daily transmission limits of 200–500 per day based on manufacturer recommendations. We included expected tag battery life according to these parameters as a covariate in the tracking duration models. All tagged turtles were released within 2 h at their capture location.

### Resident foraging regions

We assigned individual turtles to a foraging region that was defined based on prevailing currents within the Gulf of Mexico (i.e., Loop Current, Florida Current, Gulf Stream [31] and established differences in water quality between Gulf of Mexico [32] and the relatively clear, tropical waters of the Caribbean ([33]; Fig. 1). More specifically, we delineated waters between Texas and north of Naples, Florida, as the Northern Gulf of Mexico (NGOM); likewise, waters between Mexico and south of Naples, FL, were the Southern Gulf of Mexico (SGOM; Fig. 1). We delineated waters south of the Florida Straits, east of Puerto Rico, and south of Cuba as Caribbean. We delineated waters east of the Gulf Stream and north of Cuba as the Bahamas.

To assign turtles to a foraging region we examined satellite tracking data by plotting cumulative distance traveled over time (e.g., [34–36]) and examining plots to identify which date individuals reached an “asymptote”, or the point in time where distance traveled began to level out, such as a female departing a nesting beach and arriving at a foraging region (Additional file 4 a,b). We extracted the corresponding Argos location for these dates and assigned a turtle to one of the four foraging regions defined above. For turtles not reaching an asymptote (i.e., in-water captured turtles tagged at their foraging grounds that remained in the vicinity of their capture location during tracking period; Additional file 4 c-d), we selected the Argos location at the median date of tracking as the foraging site. Finally, for turtles with an increase in cumulative distance from tagging sites followed by a return to the tagging site (e.g., an in-water captured adult male turtle departing foraging grounds to a nesting beach, then returning to the foraging ground; Additional file 4 e-f), we identified the inflection point of cumulative distance from tagging site, and using data prior to that point, extracted location at the median date of that tracking period to represent the foraging site.

#### Analyses

All analyses and figures were constructed in RStudio using R version 3.6.0 [37] and species-specific foraging regions (Fig. 1) were plotted using ArcGIS 10.0 (Environmental Systems Research Institute, Redlands, California, USA). Refer to Hart et al. ([38]) for data used in analyses (below). Data exploration was carried out following the protocol described in [39] and model assumptions were verified by plotting residuals versus fitted values against all covariates. After fitting all models, we reported either pseudo-R^2^ (GLMs) or R^2^ (ordinary linear model) statistics as a measure of the model’s internal validity.

### Battery life

**Fig. 2:**
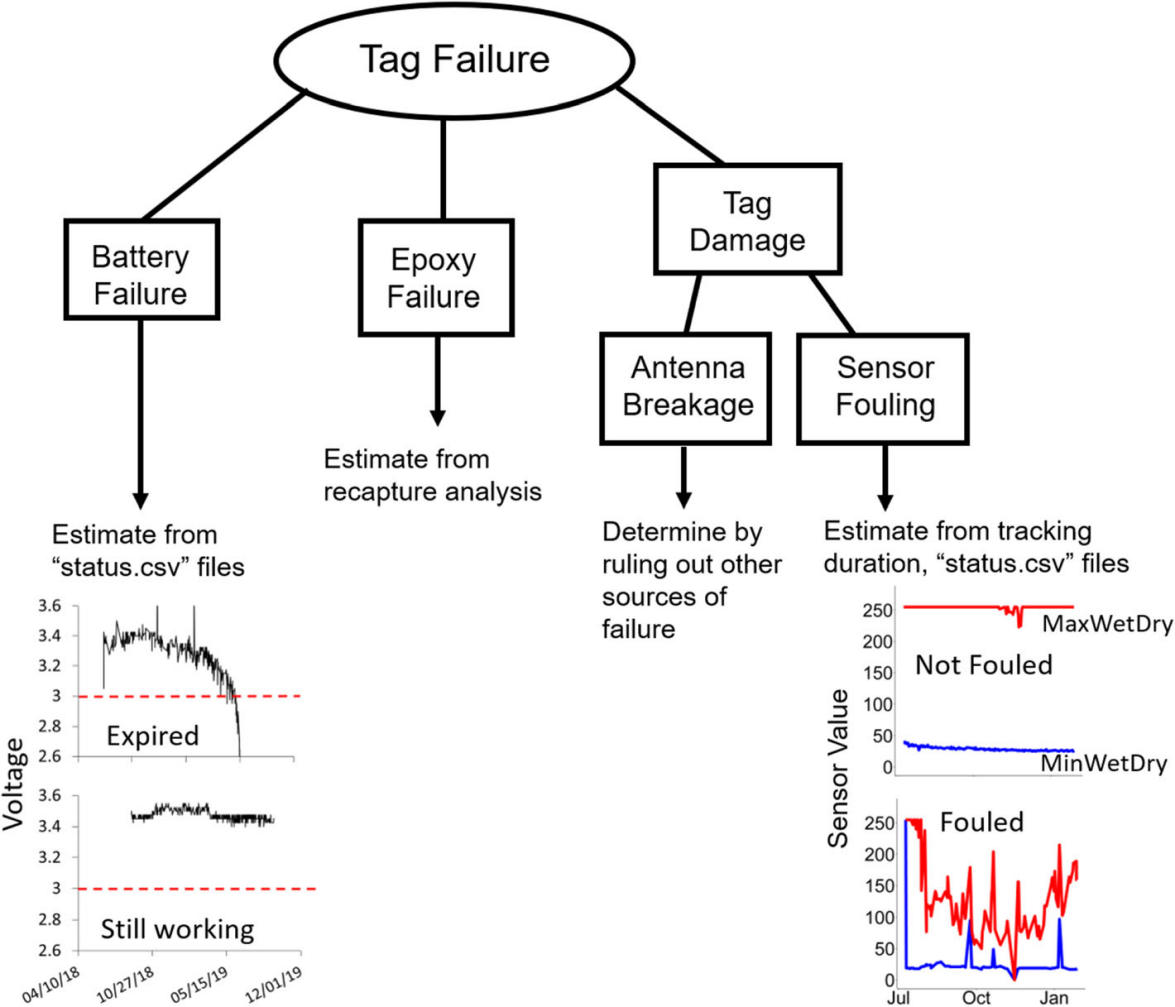
Potential causes for satellite tag failure on marine turtles and our methods for estimating each one. Our approach leveraged multiple sources of information including recaptures (epoxy failure) and auxiliary telemetry data (battery failure and sensor fouling). The data analyzed in this paper are available from the USGS ScienceBase repository: 10.5066/P9OXCKYI

To estimate battery status (i.e., working vs. expired), we examined the “status.csv” file provided from the most recent data download for each satellite tag. This status file provides non-location tag status information, such as battery voltage and readings from auxiliary sensors during each tag transmission. Full battery voltages are around 3.4 V, and tags fail when their battery voltages drop to around 3.0 V so cannot perform to capacity (Kevin Ng, Wildlife Computers, oral pers. comm., 14 February 2018). We plotted voltage of each tag over time and visually examined each plot to determine whether the battery dropped and remained below 3.0 V (Fig. 2) and categorized them as Still working/Expired. We used logistic regression with a binomial likelihood and a logit link function to estimate the percentage of tags that never had their battery expire. This was an intercept-only model (i.e., no covariates, ‘Status ~ 1’). We used the profile likelihood method (which is unbiased when parameter estimates are close to zero or 1) to generate a 95% confidence interval [40] using the generic R function ‘confint’.

### Epoxy duration

We estimated the amount of time a tag remained epoxied to a turtle by examining physical recaptures of satellite-tagged turtles, where we noted the length of time between recaptures and whether a tag was still attached. We constructed a logistic regression with a binomial likelihood and a logit link function where the presence of a tag depended upon an interaction between the number of days since it was applied and the species (i.e., ‘Status ~ NumDays * Species’). Rather than reporting the slopes estimated by this model, we reported the mean number of days that 50% of the tags remained attached, as this is a more intuitive metric of tag retention.

### Tag fouling

To determine fouling status, we examined the “status.csv” file for a subset of tags that contained data describing fouling of tag sensors. More specifically, for each tag we plotted values from the wet-dry sensor (i.e., MinWetDry and MaxWetDry values), together over time (e.g., Fig. 2); when these values approach each other, this can indicate fouling [41]. Where these two values were consistent and maintained a large distance (i.e., MinWetDry values of ~ 20, MaxWetDry values of ~ 255) for the tag’s duration, we assigned tags as “Not Fouled” [41]. Conversely, tags that contained values for MinWetDry which increased to within ~ 50–75 units of MaxWetDry at some point during the tag’s deployment or that showed these values converging toward the end of the deployment, were classified as “Fouled”. We then constructed a logistic regression with a binomial likelihood and logit link function where the fouling status of a tag depended upon the number of days since it was applied (i.e., ‘Status ~ NumDays’). As with the epoxy duration analysis, rather that reporting the slope of this line, we instead reported the mean number of days that 50% of the tags remained unfouled.

### Realized tracking duration

To estimate realized tracking duration we constructed an ordinary linear model where the length of time (days) a tag transmitted depended on the following predictors: tag model group (SPOT-location only, SPLASH-location plus depth, Fastloc GPS-location, depth and GPS), tag battery lifespan, turtle species, sex, size, and foraging region (i.e., ‘NumDays ~ Foraging_Area + species + tag type + battery life + size + sex’). Age class was incorporated in the ‘sex’ variable (i.e., female, male, immature). Expected battery life was determined based on estimates provided by the satellite tag manufacturer after taking into consideration tag model, battery size, the programming schedule and transmission limits ([38]; data available: 10.5066/P9OXCKYI).

Because turtle species in this study exhibit variation in geographic range, some species do not occur in high numbers within some foraging regions, leading to an unbalanced dataset. For example, foraging regions for loggerheads tagged in our study sites were primarily within the NGOM, SGOM, and Bahamas, whereas foraging regions for Kemp’s ridleys were primarily in the NGOM. Therefore, to explicitly evaluate species within their respective foraging regions, we built a separate ordinary linear model with tracking days as the response variable and foraging region plus species as the predictor variables.

## Results

From 2008 to 2019, we deployed 360 satellite tags on four species of marine turtles: loggerheads (*n* = 186; female *n* = 172, male *n* = 8, immature *n* = 6); green turtles (*n* = 90; female *n* = 51, male *n* = 25, immature *n* = 14); hawksbills (*n* = 42; female *n* = 36, male *n* = 1, immature *n* = 5); and Kemp’s ridleys (*n* = 42; female *n* = 34, male *n* = 6, immature *n* = 2). A subset of these tags was used for each analysis based on different criteria. Specifically, for the Battery Failure analysis, 342 tags contained data on voltage and were included in the analysis. For the Fouling analysis, 65 tags contained sensor data (i.e., MinWetDry, MaxWetDry) and could be assigned to a foraging region, thus were included in the analysis (Table 1). For the Epoxy Duration analysis, we included turtles with more than one capture event (*n* = 118). Finally, for the Tracking Duration analysis, we included the 333 turtles with satellite tags for which we could assign foraging regions (Table 2).

**Table 1:** Count of the fouling status of satellite tags on marine turtles assigned to foraging areas including the Bahamas, Caribbean, southern Gulf of Mexico (SGOM), and northern Gulf of Mexico (NGOM)

	Not Fouled	Fouled	Total
NGOM	34	17	51
SGOM	3	11	14
Caribbean*	5	2	7
Bahamas*	1	0	1
Total	43	30	
Grand Total			73

Data for NGOM and SGOM were pooled into one category, ‘Gulf of Mexico’ for analysis (Fig. 4). Asterisk (*) denotes tags excluded from fouling analyses because of small sample size

**Table 2:** Number of marine turtles with satellite tags assigned to foraging areas including the Bahamas, Caribbean, Southern Gulf of Mexico (SGOM), and Northern Gulf of Mexico (NGOM)

Species	Foraging Region					
	Bahamas	Caribbean	SGOM	NGOM	Atlantic*	Total
Loggerhead	**36**	2	**74**	**74**	2	188
Green turtle		**11**	**57**	4		72
Hawksbill	3	**34**	5			42
Kemp’s ridley			1	**32**		33
Total	39	47	137	110	2	
Grand Total						335

**Bold** values indicate data for which species-foraging region comparisons were made (Figs. 5, 6). Asterisk (*) denotes tags excluded from analyses because of small sample size

### Battery life

Analysis of battery voltage during satellite tag transmissions indicated that 14 tags out of 342 (4.1%) expired (i.e., were drained) while still attached to turtles. Thus, we interpreted this as 96% of our tags failed for some other reason before the battery died; we estimated that the probability of satellite tags remaining charged enough to successfully transmit turtle locations was 96.0% (95% CI: 93.5–97.7%).

### Epoxy duration

**Fig. 3:**
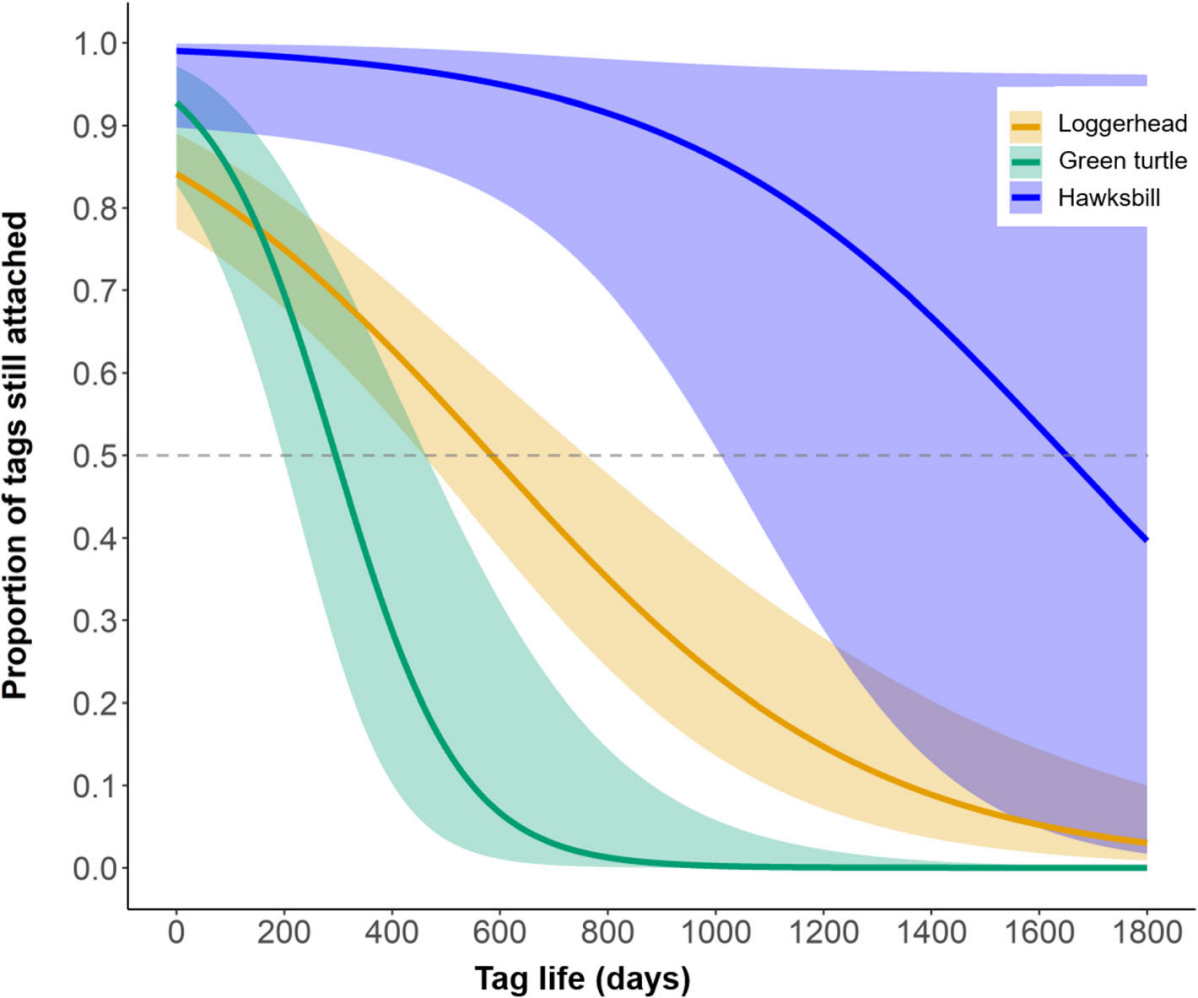
Predicted proportion of tags still attached over time (days) from the epoxy failure analysis (loggerhead, *n* data points = 214; green turtle, *n* = 88; and hawksbill, *n* = 69. Solid lines represent the mean response and shading represents 95% confidence intervals. Horizontal dashed grey line indicates the values where 50% of tags remain attached; loggerheads retain 50% of their tags for 584 days (95% CI 400–690), green turtles for 294 days (95% CI 198–450), and hawksbills for 1649 days (95% CI 995–1800). For hawksbills, we have few recaptures over 1000 days, and confidence intervals are wide after this time; however, 95% of tags remain attached for 600 days

A subset of 118 satellite tagged turtles were captured more than once (loggerheads *n* = 62, green turtles *n* = 27, hawksbills *n* = 29) and either had their tag still affixed or we noted it was not present. This dataset is primarily restricted to adult female turtles where recapture data is generated during nesting events; however, three in-water recaptures of immature turtles are also included. Across species, 47 tags (36%) remained attached for a considerably short time-frame of less than 70 days. Combining initial tagging events with subsequent recaptures resulted in 371 data points with which to fit a logistic regression (loggerheads *n* = 214, green turtles *n* = 88, hawksbills *n* = 69; pseudo R^2^ = 0.57). Results indicated that loggerheads retained 50% of their tags for 584 days (95% CI 400–690; Fig. 3). We estimated that green turtles retained 50% of their tags for 294 days (95% CI 198–450; Fig. 3). Finally, we estimated that hawksbills retained 50% of their tags for 1649 days (95% CI 995–1800; Fig. 3).

### Fouling

**Fig. 4:**
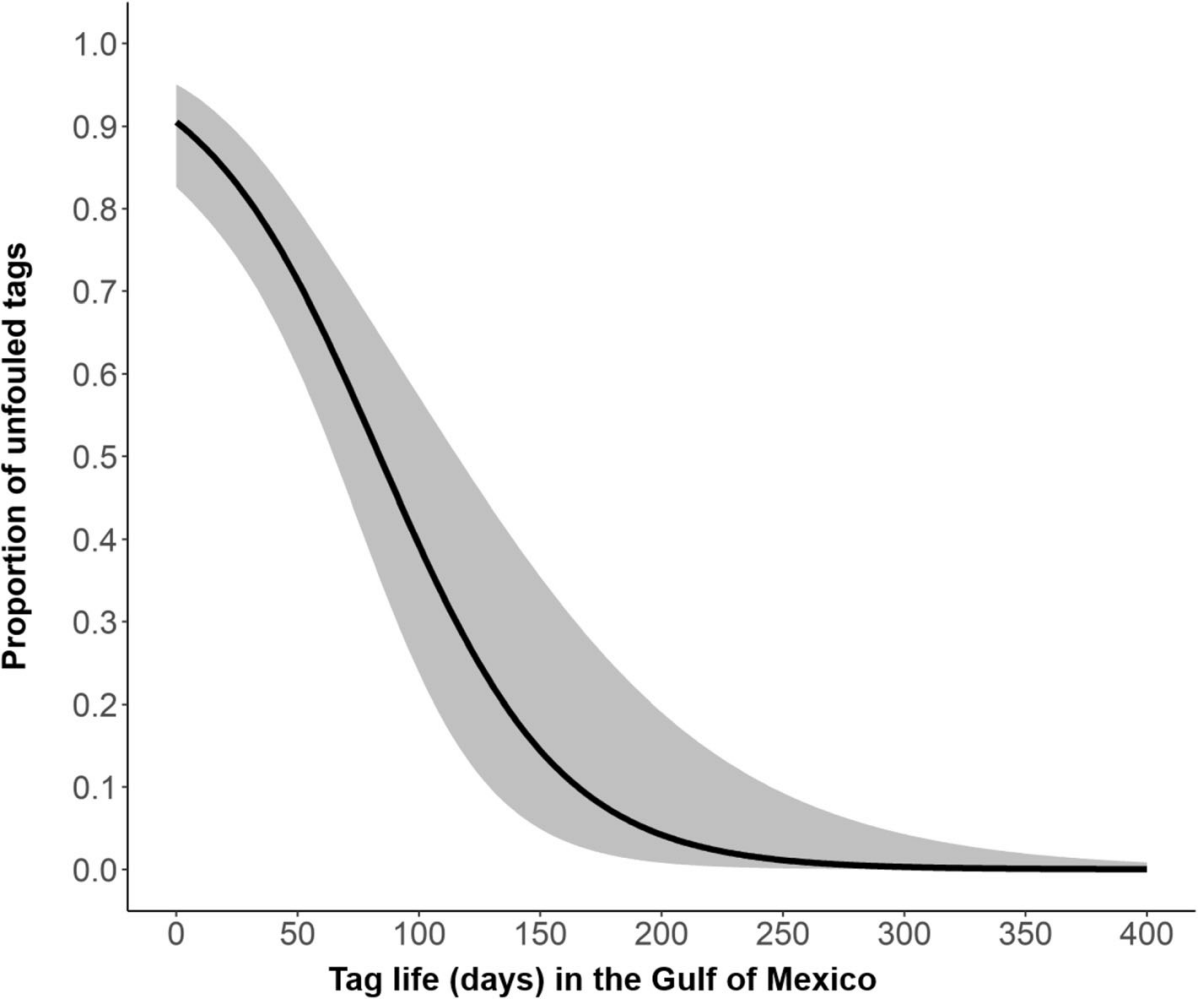
Predicted proportion of unfouled tags over time (days) in the Gulf of Mexico from the fouling analysis (*n* = 65 tags: unfouled *n* = 37, fouled *n* = 28). Solid line represents the mean response and gray shading represents the 95% confidence interval

Of 73 satellite tags with available data for analysis (Table 1), the majority were attached to turtles foraging in NGOM (*n* = 51) and SGOM (*n* = 14) rather than the Caribbean or Bahamas, therefore we restricted our comparison to the Gulf of Mexico (*n* = 65 tags). Within the Gulf of Mexico, 28 of 65 satellite tags became fouled (43%); such fouling did not always result in the complete loss of transmissions and derived locations. We estimated that in the Gulf of Mexico, across species, after 83 days, 50% of satellite tags (95% CI: 70–120) became fouled; pseudo R^2^ = 0.44, Fig. 4, Additional file 3). Examination of the raw data indicated that of tags that fouled, 18 were on loggerheads, 8 were on green turtles, and 2 were on Kemp’s ridleys.

### Realized tracking duration

**Fig. 5:**
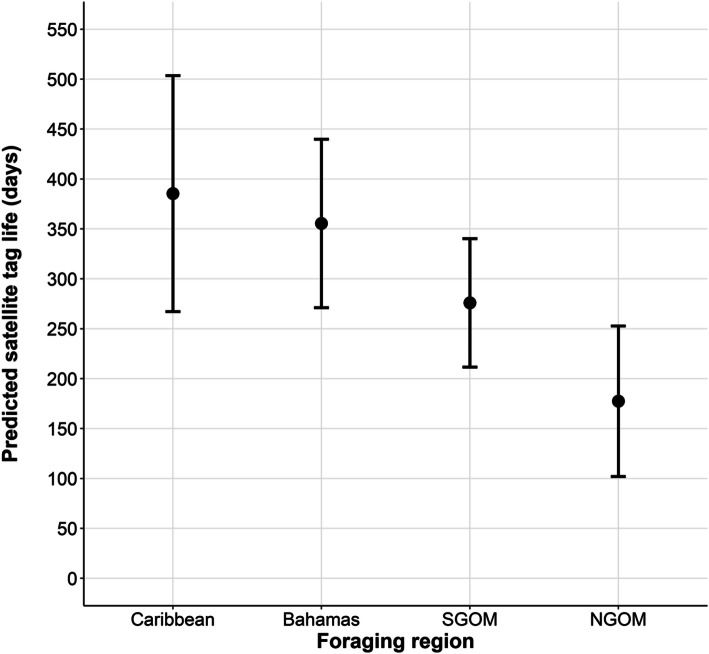
Predicted length of time (days) that satellite tags attached to marine turtles will transmit within foraging regions (Caribbean, Bahamas, Southern Gulf of Mexico (SGOM) and Northern Gulf of Mexico (NGOM) based on length of time each tag (*n* = 333) transmitted. Solid black circles represent mean estimates and lines represent 95% confidence intervals

**Fig. 6:**
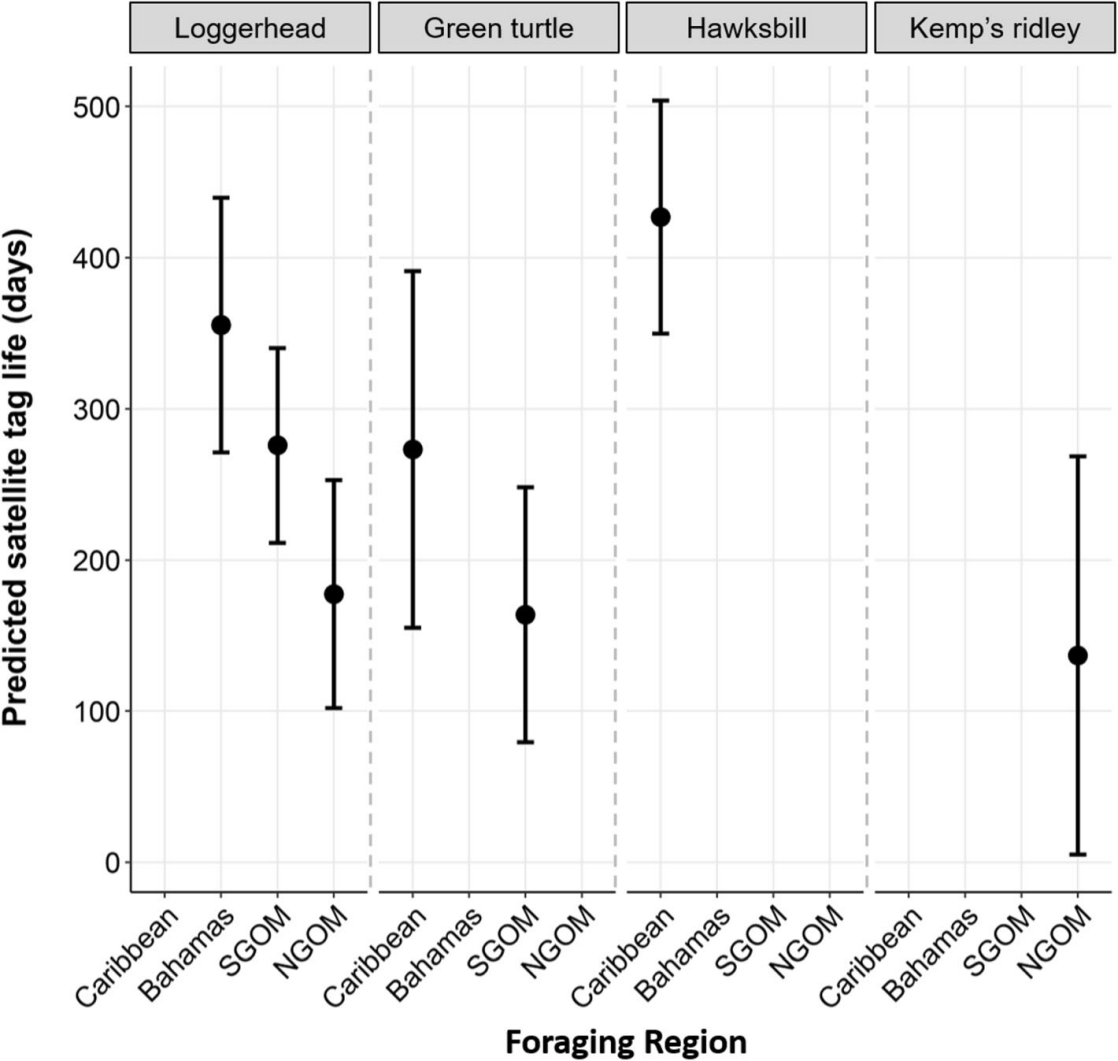
Predicted length of time (days) that satellite tags transmit on marine turtles within foraging regions. Solid black circles represent mean estimates and lines represent 95% confidence intervals. The southern and northern Gulf of Mexico are abbreviated as SGOM and NGOM, respectively. Some species are not prevalent in all foraging regions and thus we make no predictions there (e.g., hawksbills in the SGOM). Our findings show that both species and location are important in determining realized tracking durations

Our realized tracking duration model included 333 turtles (loggerheads = 186, green turtles = 72, hawksbills = 42, Kemp’s ridleys = 33, Table 2; sex: female = 284, male = 33, immature = 16) with a total of 86,889 tracking days. Based on model estimates, the length of tracking duration was strongly influenced by foraging region and species (Table 3, Fig. 5; R^2^ = 0.20). The shortest durations were observed in the NGOM, followed by the SGOM, Bahamas, and then the Caribbean. Model-estimated tracking durations, using loggerheads as the reference category, were as follows: Bahamas (356 days, 95% CI 271–440), Caribbean (385 days, 95% CI 267–504), SGOM (276 days, 95% CI 212–340), and NGOM (177 days, 95% CI 102–253; Figs. 5 and 6). Using SGOM as the reference category, the shortest durations were observed from Kemp’s ridleys (NGOM: 137 days, 95% CI 5–269), followed by green turtles (SGOM: 164 days, 95% CI 79–248), loggerheads (NGOM: 177 days, 95% CI 102–253), and then hawksbills (Caribbean: 427 days, 95% CI 350–504; Fig. 6).

**Table 3:** Model structure and rankings examining predicted number of days satellite tags are predicted to transmit for marine turtles

	Estimate	Std. Error	t-statistic	*p*-value
Intercept	238.29	208.99	1.14	0.255
Foraging Region (Caribbean)	29.90	62.58	0.48	0.633
Foraging Region (NGOM)	−178.07	50.26	−3.54	0.000
Foraging Region (SGOM)	−79.54	42.43	−1.88	0.062
Species (*C. mydas*)	−112.20	39.00	−2.88	0.004
Species (*E. imbricata*)	41.49	60.29	0.69	0.492
Species (*L. kempii*)	−40.62	61.33	−0.66	0.508
Tag Model (SPLASH)	−23.72	74.89	−0.32	0.752
Tag Model (SPOT)	−21.27	89.84	−0.24	0.813
Battery Capacity	0.09	0.16	0.56	0.576
Size	0.76	1.70	0.45	0.654
Sex (Males)	64.97	43.36	1.50	0.135
Sex (Immature)	190.02	73.60	2.58	0.010

Dataset is female-biased and sample size for immature turtles is small (females = 284, males = 33, immature = 16); refer to Tables 1–2 for sample size among other categorical variables. The intercept (i.e., reference category) is comprised of female loggerheads in the Bahamas with GPS tags

**Fig. 7:**
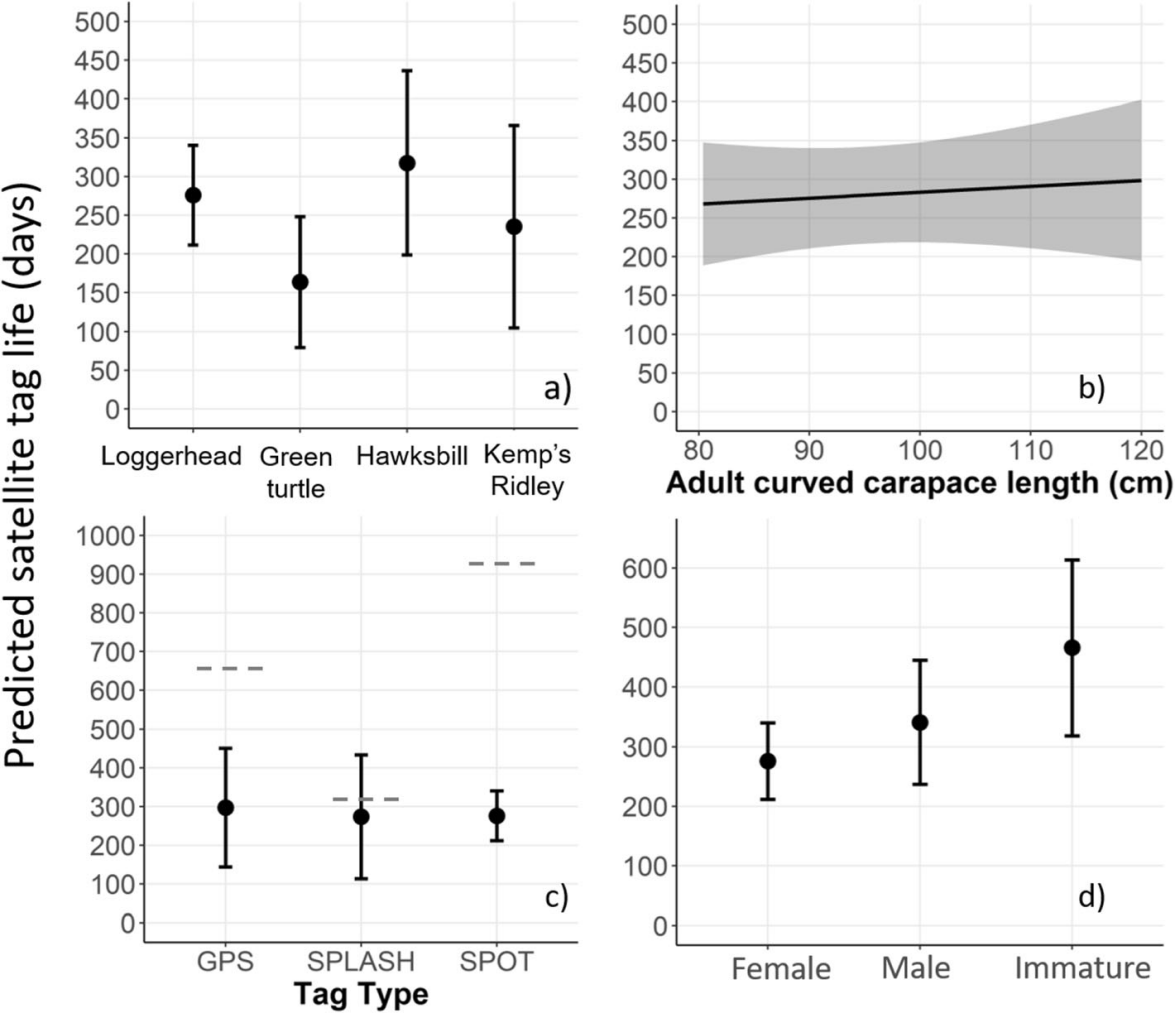
Predicted length of time (days) that satellite tags transmit on a) marine turtles b) by size, c) by tag model type, and d) by sex. Solid black circles represent the mean and lines represent 95% confidence intervals; dashed lines in c) represent mean expected battery life for tag types, from manufacturer calculations. Our findings indicate that adult size, tag type, and sex are not important in determining realized tracking durations; instead only species and foraging region (Fig. 6) drive observed patterns in tracking durations

Of 333 satellite tags, most (75%) were SPOT models (*n* = 250) compared to SPLASH tags (*n* = 71) or Fastloc GPS tags (*n* = 12; Additional file 2). Across all tag types, 21 tags of 333 (6.3%) transmitted longer than expected (i.e., exceeded expected battery life). On average, 38.1% of SPOT tags reached their expected battery life and similarly, 44.7% of SPLASH and 32.2% of GPS tags reached their expected tag life. Satellite-tag specific manufacturer estimates of battery life (accounting for tag model and programming schedules) varied from 320 to 914 days for SPOT tags, from 165 to 485 for SPLASH tags, and 485 to 1007 days for GPS tags. However, estimated battery life did not have a significant effect on realized tracking duration, corroborating our findings from the previous sections (β = 0.09, *p* = 0.576; Table 3). Similarly, tag model did not have a significant effect on tracking duration. With GPS tags as the reference category, neither SPOT (β = − 21.27, *p* = 0.81; Table 3) nor SPLASH (β = − 23.72, *p* = 0.75; Table 3) tags differed in tracking duration. More specifically, tag model types transmitted for a similar duration of time, and on average, SPOT tags transmitted for 276 days (95% CI 212–340), SPLASH tags transmitted 274 days (113–434), and GPS tags transmitted for 297 days (144–451; Table 3, Fig. 7). Likewise, size (β = 0.76, *p* = 0.65) did not have a significant effect on tracking duration of adult turtles, and sex was important (β = 190.02, *p* = 0.01; Table 3), with females tending to transmit for shorter periods; the patterns for juveniles was likely driven by small sample size of the immature age class (Fig. 7).

## Discussion

Here, we provided a robust analysis of a long-term, large-scale, multi-species satellite tracking dataset focused on marine turtles to decipher what influenced tracking duration and identify likely causes of tag and transmission failures. In a rare exploration of what influences satellite tag functionality, we found that tag battery life was not the limiting factor, rather it was the environment. Our findings suggest a mechanism for tag failure (i.e., tag damage) that will inform future research questions of the biologging community. Specifically, we observed a pattern among foraging regions, where tracking duration varied latitudinally, decreasing in order from the Caribbean and Bahamas (~ 356–385 days) to the SGOM (~ 276 days), and NGOM (~ 177 days). Average battery failure rate for tags was ~ 4%, indicating that 96% of tags failed for another reason before batteries drained. For all species, tags tended to remain attached for at least 1 year. Notably, the estimated rate of tag fouling reached 50% after 83 days in the Gulf of Mexico, and despite tags transmitting from sites in the Bahamas and Caribbean, few became fouled in these regions. We suggest that because the incidence of battery failure was so low, and recapture information indicated that tags remained affixed to turtles for at least 1 year, that most tag failures were caused by tag damage (sensor fouling and/or antenna breakage) rather than battery or epoxy failure.

### Foraging regions drive ultimate tracking duration patterns

We found that the number of days satellite tags transmitted varied by foraging region, where marine turtles spend most of their life. Further, in the Gulf of Mexico foraging region, 50% of tags fouled rapidly (i.e., within 3 months), consistent with previous research indicating that barnacle settling rates in the NGOM are elevated [42].

We suggest variation in tag transmissions across foraging regions was in part driven by regional differences in water quality, directly related to the Gulf of Mexico’s Loop Current that is part of the Gulf Stream system of the Atlantic Ocean. Characteristics of the Loop Current and its anticyclonic eddies have been studied with satellite-tracked drifters and remote sensing, and indicate the powerful influence of this current on water circulation in the Gulf of Mexico [43]. Briefly, the Loop Current enters the Gulf of Mexico northward from the Yucatan Peninsula, moves clockwise, and exits through the Florida Straits between Cuba and Key West, Florida [31]. This pattern of water flow creates several different “Ecoregions” in the Gulf of Mexico, whereby the NGOM Ecoregion is distinct from the SGOM with respect to specific habitat types, bathymetry, eutrophication, hypersalinity [44] and anthropogenic influences [45]. Anecdotally, we tracked two loggerheads foraging in the Atlantic and compared them to turtles foraging within the NGOM. The Atlantic foraging region is at approximately the same latitude as NGOM foraging grounds (Fig. 1), yet these tags transmitted longer (208 and 479 days) than the average for NGOM tags (~ 177 days [38];), likely because the Atlantic does not have the same eutrophic water conditions as the NGOM. Although we have not determined if fouling was extensive enough to stop tag transmissions, we suggest that nutrient-rich waters may be responsible for fouling of tags in our study and is likely responsible for the shorter tracking durations we observed in the Gulf of Mexico. Studies investigating the degree to which biofouling influences transmission signal strength could inform and improve future tracking work.

Ultimately, our results indicate that tags transmit for a shorter duration in the NGOM foraging region because of both fouling of tags and potential species-specific variation in behavioral aspects (e.g., hiding under structures). We suggest that where turtles live and take up residence affects aspects of tag damage, both physical (i.e., antenna breakage) and ecological (i.e., tag fouling; see Additional file 3). Frick et al. [46, 47] documented significant loads of epibionts living on turtles, thus anti-fouling paint is one solution for minimizing rates of fouling in tracking studies. We have recently begun applying anti-fouling paint to all tags across all projects in order to deter growth of marine organisms ([6]; see Additional file 1). Future studies to quantify the effect of antifouling paint on tracking durations would be valuable. However, in some areas barnacle settling rates are naturally elevated (e.g., Ship Shoal, LA [42]). Thus, improvements in anti-fouling technology beyond paint are particularly important for tags deployed in the NGOM. Notably, attempts during this study to modify tags deployed in NGOM by increasing the 3-dimenstional aspect (i.e., having cone shaped washer/sensor on tags) to facilitate easier scraping of epibionts by turtles have not prevented rapid fouling rates on tags transmitting in the NGOM (mean number of transmission days for tags with a cone was 149 days (*n* = 83) and tags without a cone transmitted an average of 185 days (*n* = 27). Development of additional physical ‘windshield washers’ on sensors may reduce epibiont loads, and creation of miniature sensors for antennas to relay data on their status (i.e., broken, fouled) may further improve satellite tag function.

### Species-specific variation in tracking duration and tag attachment

Our findings are consistent with that of previous acoustic tagging research on marine turtles that showed shorter tag retention times for green turtles compared to hawksbills of similar (juvenile) sizes. Specifically, green turtle tag retention rates in a Caribbean study were 1/7th that of hawksbills [48]. Although the attachment methods are different for acoustic versus satellite tags, this comparison underscores the difficulty of obtaining long tracking durations on green turtles with external epoxy attachments. Notably, we observed long tracking durations on species with sometimes significant epibiont loads (i.e., loggerheads) compared to green turtles (Fig. 6), underscoring the importance of foraging region on fouling and realized tracking durations. Perhaps contributing to increased epibiont loads on loggerheads are their more sedentary behavioral tendencies [49, 50].

These results underscore that there can be species-specific differences in tag performance, even within taxonomic families. Studies that examine tag fix rates (the proportion of attempted location fixes that are successful) are commonly concerned with bias driven by habitat conditions [51]. This implies that fix rates are habitat-dependent and typically ignores the role that species-specific traits (e.g., behavior, shell hardness) may play. For example, Smith et al. [52] studied the fix rates of GPS tags implanted in Burmese pythons (*Python bivittatus*) and assumed that their conclusions were applicable to all large constricting snakes (families Boidae and Pythonidae). Other studies have compared the impact of tag attachment on different species; the same GPS attachment method that was appropriate for Black-backed Gulls (*Larus fuscus*) greatly reduced apparent survival of Great Skuas (*Stercorarius skua*) [53]. As we have shown here, tag performance can vary greatly within a taxon, and in some cases species-specific tagging protocols may be necessary. Furthermore, comparative study designs seeking ecological inference from the spatial patterns of different closely related species (e.g., [54, 55]) should take differential tag performance into consideration to avoid biasing their conclusions.

### Implications for future study design

Establishment of more precise estimates of mean tracking duration specific to a particular study site or species is helpful for guiding expectations for the amount of return data. Further, considerations of tag model choice and timing of tagging are important and carefully matching study objectives to tag choice is critical. Thus, if a research priority is to track a nesting turtle from breeding to foraging regions, tags must function for at least several months to collect enough data to separate error in location points with actual movement (i.e., migration).

Our results also answer the call of Jones et al. [11] to consider critically our attachment techniques; we found that it does not take much adhesive (Additional file 1) to obtain long tracking durations for any of our low-profile, low-drag tags. To fill key knowledge gaps, studies must match study design and realistic expectations of tag performance. Our dataset included tags that remained attached for as little as 30 days to those lasting as long as 1653 days, and included data on turtles across life stages and across several species for which there is limited tracking data globally (i.e., males and immature turtles, see [3, 5, 56, 57]). Thus, our results contribute towards filling data gaps for imperiled sea turtles captured in both developmental and foraging areas. Further, we hope that our expected tracking durations and predicted tag attachment duration estimates (Fig. 3), along with our attachment protocol, will be useful for both permitting agencies and funding parties in matching expected project results to what is feasible in field studies, ultimately achieving more conservation dividends for these imperiled marine turtles [15]. In particular, the type of information we present here can inform managers charged with decisions on the tradeoffs of collecting more data versus time to collect it [58] and the value of animal movement for management planning [59]. Without a clear picture of what to expect for actual tracking durations from various tags, researchers may mismatch research questions and study design, thus rendering them unable to effectively translate their data into useful conservation approaches.

### Summary

Biologging tools continue to play a key role in determining marine animal movement patterns, including data on timing of migrations, spatial extent of corridors used, and locations where animals concentrate their home ranges [6, 60]. Thorough examination of robust tracking datasets is critical to improve data provided by these tools (e.g., satellite tags). Improving data quality is particularly important because these tools are frequently used on imperiled species, some of which (like marine turtles) may breed only every 1–3 years. Most tag failures across all marine turtle species in our study were caused by tag damage, either consisting of sensor fouling or antenna breakage, which are currently difficult to tease apart. However, sensor data are available and future sensor development distinguishing between types of tag damage would improve the quality of tracking data. Specifically, increasing regular messaging of diagnostic data, improving design and placement of sensors, and creating more physical protection for robust antennas could improve tracking science.

Tag manufacturers make their best estimates for tag ‘life’ based on battery capacity. But real-world factors such as programming schedules, animal behavior, and environmental conditions affect the realized tracking durations across species. Our results indicate that provided tags remain attached to animals and intact, they have enough battery to meet or exceed manufacturer recommendations. Future improvements to sensors to send additional data reflecting presence and condition of tags and antennas, in conjunction with innovations such as miniaturized batteries, longer-logging accelerometers, and on-board data-processing algorithms can improve our understanding of animal movement patterns and what drives them, and ultimately help researchers explore life in the wild when animals are not directly observable.

## Supplementary Information

**Additional file 1.** Satellite tag attachment protocol for hard-shelled sea turtles.

**Additional file 2.** Number of satellite tags deployed at different tagging sites during each year. Refer to main text Fig. 1 for generalized tagging locations. Size of bubble scaled by sample size, and abbreviations for tagging sites are as follows: Belle Pass, Louisiana = BPLA; Gulf Shores, Alabama = AL; various northern Gulf of Mexico in-water sites in Louisiana, Mississippi, northwest Florida = NGOM; Dry Tortugas National Park = DRTO; Everglades National Park/Biscayne National Park = ENP; Buck Island Reef National Monument = BIRNM. Satellite tag models were comprised of SPOT (*n* = 250; models 244A, 293A, and 375A), SPLASH (*n* = 71; models 284A, 296F, 297F, 309A, 238A) and GPS (*n* = 12; models 296F, 344E, 238A, 385A).

**Additional file 3.** Example of SPOT tag fouling by marine organisms on a loggerhead turtle (*Caretta caretta*) in the northern Gulf of Mexico. Tag was attached a) June 13th and b) 25 days later (July 8th, 2013) the tag is covered primarily with barnacles, although the antenna is visible. Photograph by the U.S. Geological Survey.

**Additional file 4.** Methodology used to assign marine turtles to foraging regions by plotting satellite tag location data (panels a, c, e) and plotting cumulative distance traveled over time for each individual (panels b, d, f). Visualizing these data can indicate the point in time where distance traveled begins to level out/reach an asymptote (i.e., red dashed lines). We designated foraging sites near the asymptote. Examples include a) a nesting female departing the Dry Tortugas and arriving at a foraging site in the Bahamas, c) an in-water captured male resident of the Dry Tortugas, who made some looping movements away from, and then back to, the Dry Tortugas, and e) a male migrating from the waters offshore of Cancun, Mexico presumably to breed, then exhibiting return migration to the foraging site at the Dry Tortugas. Grey shaded boxes in panels b and f indicate migration intervals; no migration is occurring in panel d for this resident turtle.

## Data Availability

The data analyzed in this paper are available from the USGS ScienceBase repository: 10.5066/P9OXCKYI.

## Abbreviations

Fig.: Figure
NGOM: Northern Gulf of Mexico
SGOM: Southern Gulf of Mexico
GPS: Global positioning system
PIT: Passive integrated transponder
PTT: Platform terminal transmitter
