# Supplementary material for: Drivers of realized satellite tracking duration in marine turtles
Source: Mov Ecol. 2021 Jan 5;9:1. doi: 10.1186/s40462-020-00237-3 (PMC7786511; doi:10.1186/s40462-020-00237-3)
Supplement: Supplementary file 1 — Additional file 1. Satellite tag attachment protocol for hard-shelled sea turtles. [file 40462_2020_237_MOESM1_ESM.docx]

**Additional file 1. Satellite tag attachment protocol for hard-shelled sea turtles.**

**Materials needed**

- 1 Satellite tag
- 1 grease syringe
- 1 rubber stopper
- Mixing cup (e.g., clean circular yogurt container or similar)
- Superbond epoxy [Brand: Superbond™; Epoxy: Fast Curing Agent, EA-089B3 and Resin: EA-089A]
- Wooden tongue depressors (5)
- Paper towels
- Sandpaper (80 grit)
- Electrical tape
- 91% isopropyl alcohol
- Screwdriver
- Paint scraper
- Water source (fresh or sea)
- Gloves
- Tarp
- Watch/timer
- Trash bag/container

**Overview: Tag preparation and epoxy**

TAG PREP/ANTIFOULING

Before leaving for the field, apply anti-fouling paint to the tags to deter growth of marine organisms on tags and sensors. Available options are Interlux – Micron 66 Antifouling Paint, Aquaguard Waterbase Bottom Paint, Pettit Paint Hydrocoat and more recently, Propspeed Lightspeed Underwater Light Coating. While applying anti-fouling paint (<https://wildlifecomputers.com/our-tags/extras/anti-fouling/>), cover and protect each sensor (i.e., small silver disc, usually on top of the tag or along the sides) with a small piece of electrical tape. For long-term storage, tags should be kept refrigerated to preserve battery.

STORAGE OF EPOXY IN THE FIELD

The curing of epoxy is an exothermic reaction (i.e., gives off heat). The rate at which it gives off heat depends on starting temperature, volume of mixed epoxy, and ambient temperature. When working at night on nesting beaches, temperature does not seem to greatly influence the curing process. However, during the day, although our epoxy is a cool-setting epoxy, warm temperatures can affect heat produced by mixing the two-parts of the epoxy. Keep both the epoxy and the curing agent (i.e., parts A and B) in a cooler during daytime tagging; do not bury in ice, just keep it cool and out of direct sunlight.

VOLUME OF EPOXY

After applying a tag to a turtle, avoid the temptation to believe more epoxy is better. The epoxy that will hold the tag in place is that between the tag and the turtle’s carapace; additional epoxy applied thickly around (or on top of) the tag does not make the tag adhere better. While additional epoxy can be strategically applied to improve the hydrodynamic properties of the tag, or to protect against something hard the turtle may come in contact with, the total volume of epoxy and tag footprint should be minimized to prevent excessive heat felt by turtles during the curing process. Turtles tagged during the day should be shaded throughout the curing period (we use wet towels to cover the turtle’s head and areas of the carapace behind the tag).

**SAT TAG APPLICATION**

**Step 1: Prepare the tag**

Complete all programming, use the grease syringe to drop a small dab into the comm port, then plug the port w/ rubber stopper.

**Step 2: Prepare the turtle’s carapace for tag attachment**

Prior to tag application, remove any barnacles and sand down the tag application area (typically the second vertebral scute on carapace) using 80 grit wet/dry sandpaper. Continually rinse the area being sanded to minimize accumulation of shell debris on the sandpaper. After sanding is complete, the area should be thoroughly cleaned with 91% isopropyl alcohol and then dried completely; dry with a dabbing motion to minimize fiber residue left by paper towels. The alcohol/drying process should be repeated 3 times. At this time, the tag application area should not be touched as oils from the skin may reduce tag adherence.

Note: The most important aspect of a strong tag application is a clean, dry starting surface. Therefore, if it is raining, set up a tarp or use a rain jacket to shield the tag application area during attachment and until the epoxy has cured (~ 1 hour).

**Step 3: Prepare the tag for application**

To prevent epoxy from dripping on sensors, temporarily apply electrical tape over each sensor, as well as any recessed pins or depth sensors associated with tags that record dive information. In the event epoxy is dripped on a sensor, immediately wipe off with a paper towel and 91% isopropyl alcohol; if it has hardened, use sandpaper and 91% isopropyl alcohol to clean the sensor.

**Step 4: Epoxy mixing**

Epoxy should be mixed in a clean, disposable container (e.g., a well-cleaned yogurt cup, wiped with isopropyl alcohol before using). Each mixing container is used once and then discarded. To apply a standard satellite tag (i.e., Wildlife Computers SPOT and SPLASH tags), we mix approximately 1½ tablespoons (e.g., a golf ball size volume) each of the epoxy (A) and curing agent (B).

- Scoop out a golf ball sized volume of A w/ one wooden tongue depressor; discard the wooden stick.
- Scoop out an equivalent golf ball sized volume of B w/ a second (new) wooden tongue depressor; discard this wooden stick (see Fig. S1A). Separate sticks are used to avoid contaminating the reserves of one material with the other. [Note: The epoxy and curing agent should be mixed at a ratio of 1:1 by volume.]
- Next, get a 3^rd^ new wooden tongue depressor and start mixing the epoxy. (Fig. S1B)

As soon as mixing begins, have someone on the team start a countdown clock to keep track of the epoxy cure time. The epoxy and curing agent should be thoroughly and constantly mixed for 3 minutes. Scrape the inside edges and bottom of the cup to achieve a thorough mix.


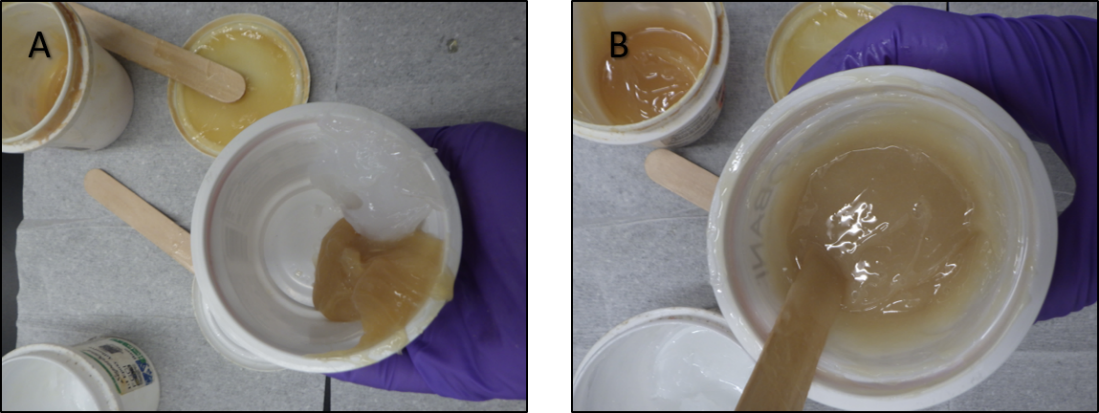


Figure S1. Equal parts of epoxy and curing agent are placed in a clean disposable container (A) and then thoroughly mixed (B), scraping the sides of the cup to ensure a complete and uniform mixture. Photographs by the U.S. Geological Survey.

**Step 5: Epoxy application**

Turtle

At this point, anyone handling the epoxy, tag, or turtle should be wearing gloves. Apply a thin layer of the mixed epoxy to the turtle’s carapace, at the tagging location (typically the second vertebral scute; Fig. S2A). For maximum bond, a layer of epoxy approximately 1-2 mm thick is required between the turtle and the tag. Flatten the epoxy so it matches the dimensions of the satellite tag, taking care to avoid trapping air bubbles between the turtle and the epoxy footprint (Fig. S2B).

When the epoxy has been spread to ensure complete coverage beneath the tag footprint, apply slightly more epoxy to the center of the footprint so that when the satellite tag is pressed into place, excess epoxy is forced out along the edges, such that that no air gets trapped beneath the tag.

Tag

Have one person carefully peel off the sterile paper on the bottom of the satellite tag; do not touch the newly exposed surface. “Butter” the bottom (all the way to the edge) of the tag with a thin layer (i.e., 1-2 mm) of epoxy to ensure complete contact (Fig. S2C-D). Turn the tag over and align it where it will ‘meet’ the epoxy on the turtle. Slowly press the tag into the epoxy footprint on the turtle (Fig. S2E-F); orient the tag with antenna pointed towards the head of the turtle to minimize any damage to the antenna during possible courtship and maximize area of antenna exposed when turtles surface. As the tag is pressed into the footprint, rotate slightly as needed to maintain a centered position. Firmly press the tag to secure it in place and then do not move the tag again; this sometimes requires holding the tag in place for 10-15 seconds to ensure it doesn’t slide if the surface is not level or if the turtle is not resting quietly.

Forming the epoxy around the tag

Use excess epoxy that squeezes out from under the tag to create a hydrodynamic shape (Fig. S2G) around the tag ‘base’. Using a new tongue depressor (held at ~45 degree angle), gently form the epoxy around the lower sides of the tag in a bead (e.g., as with caulk on the seam between backsplash and a kitchen countertop), using long strokes (not short ones) to create a smooth surface, adding additional epoxy as needed (Fig. S2G-H). After 10-15 minutes, check for tackiness. Once the epoxy starts getting les tacky, you can use a wet gloved finger to smooth the epoxy. After 10-15 minutes, check for tackiness. Once the epoxy starts getting les tacky, you can use a wet gloved finger to smooth the epoxy.

Covering the rubber stopper over the sat tag communications port w/ epoxy

Break another tongue depressor lengthwise to create a thinner stick and drop or dab a small amount of epoxy over the rubber stopper to cover it. Be very careful NOT to get epoxy on tag sensors. As stated earlier, you may want to put electrical tape temporarily over the sensors but remember to remove tape after epoxy cures and prior to release (Fig. S2I). If epoxy gets on sensors, remove with paper towel and alcohol or, if epoxy has hardened on sensors, sandpaper it off.

Epoxy drying and curing time

Turtles can typically be released 45-60 minutes after mixing the epoxy and affixing the tag to the carapace. Curing time may be longer in cool, damp, or low-humidity conditions. Make sure the tag’s LED light is flashing (i.e., transmitting) before releasing the turtle, however, note that flashing will not be visible if the tag has been painted with anti-fouling paint; in this case, a Wildlife Computers test pinger can be used to listen for a transmission tone to ensure tag is on and transmitting. Turtles can be released when the epoxy is hard enough NOT to show a fingernail mark if you press into it. The epoxy will continue to cure and strengthen for 24 hours, but immersion in water during this time will neither slow the process nor weaken the final bond.

**
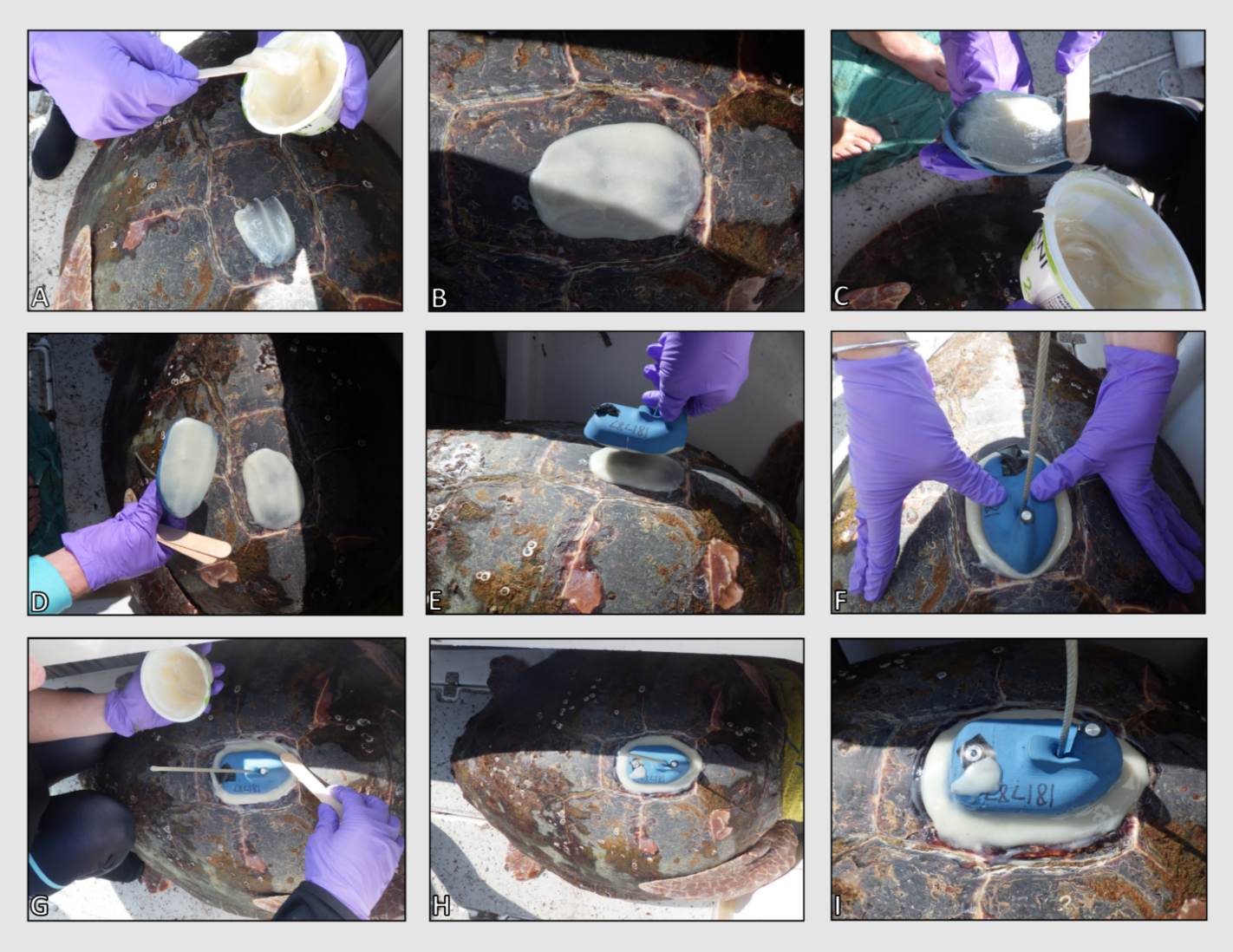
**

Figure S2. Satellite tag application on a loggerhead turtle depicting A-B) epoxy placement on carapace, C-D) epoxy application on tag (layer 1-2 mm thick), E-F) tag application, G) establishing a hydrodynamic epoxy footprint, and H-I) completed process with communications plug epoxied and tape removed from sensor. Tag in figure is painted with anti-fouling paint. Photographs by the U.S. Geological Survey.
