# Supplementary material for: Drivers of realized satellite tracking duration in marine turtles
Source: Mov Ecol. 2021 Jan 5;9:1. doi: 10.1186/s40462-020-00237-3 (PMC7786511; doi:10.1186/s40462-020-00237-3)
Supplement: Supplementary file 2 — Additional file 2. Number of satellite tags deployed at different tagging sites during each year. Refer to main text Fig. 1 for generalized tagging locations. Size of bubble scaled by sample size, and abbreviations for tagging sites are as follows: Belle Pass, Louisiana = BPLA; Gulf Shores, Alabama = AL; various northern Gulf of Mexico in-water sites in Louisiana, Mississippi, northwest Florida = NGOM; Dry Tortugas National Park = DRTO; Everglades National Park/Biscayne National Park = ENP; Buck Island Reef National Monument = BIRNM. Satellite tag models were comprised of SPOT (n = 250; models 244A, 293A, and 375A), SPLASH (n = 71; models 284A, 296F, 297F, 309A, 238A) and GPS (n = 12; models 296F, 344E, 238A, 385A). [file 40462_2020_237_MOESM2_ESM.docx]

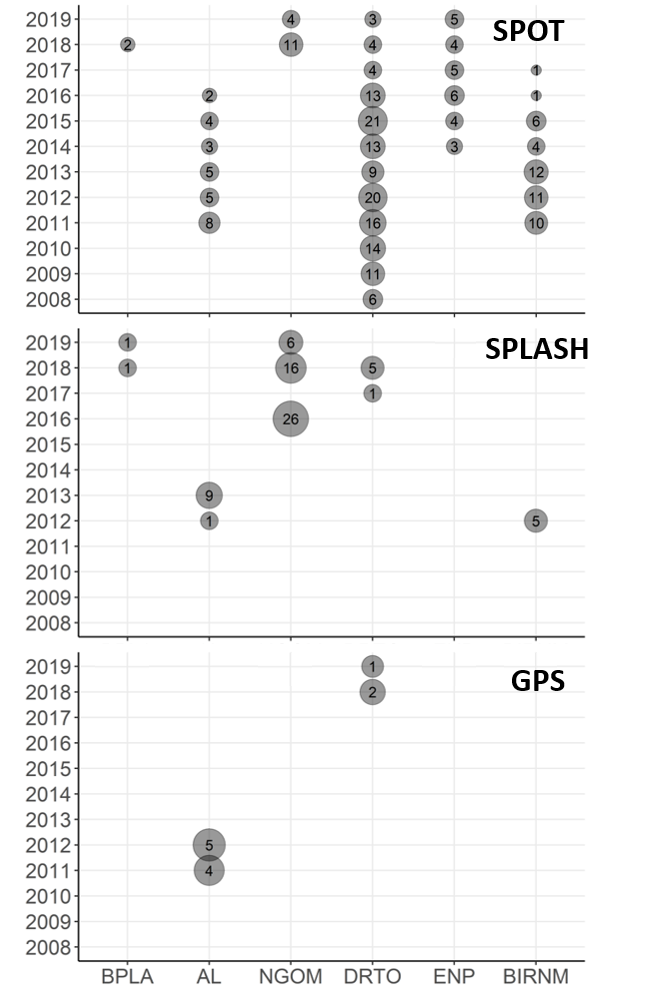
Additional file 2. Number of satellite tags deployed at different tagging sites during each year. Refer to main text Fig. 1 for generalized tagging locations. Size of bubble scaled by sample size, and abbreviations for tagging sites are as follows: Belle Pass, Louisiana = BPLA; Gulf Shores, Alabama = AL; various northern Gulf of Mexico in-water sites in Louisiana, Mississippi, northwest Florida = NGOM; Dry Tortugas National Park = DRTO; Everglades National Park/Biscayne National Park = ENP; Buck Island Reef National Monument = BIRNM. Satellite tag models were comprised of SPOT (n = 250; models 244A, 293A, and 375A), SPLASH (n = 71; models 284A, 296F, 297F, 309A, 238A) and GPS (n = 12; models 296F, 344E, 238A, 385A).
