# Supplementary material for: Drivers of realized satellite tracking duration in marine turtles
Source: Mov Ecol. 2021 Jan 5;9:1. doi: 10.1186/s40462-020-00237-3 (PMC7786511; doi:10.1186/s40462-020-00237-3)

Additional file 3. Example of SPOT tag fouling by marine organisms on a loggerhead turtle (*Caretta caretta*) in the northern Gulf of Mexico. Tag was attached a) June 13^th^ and b) 25 days later (July 8^th^, 2013) the tag is covered primarily with barnacles, although the antenna is visible. Photographs by the U.S. Geological Survey.


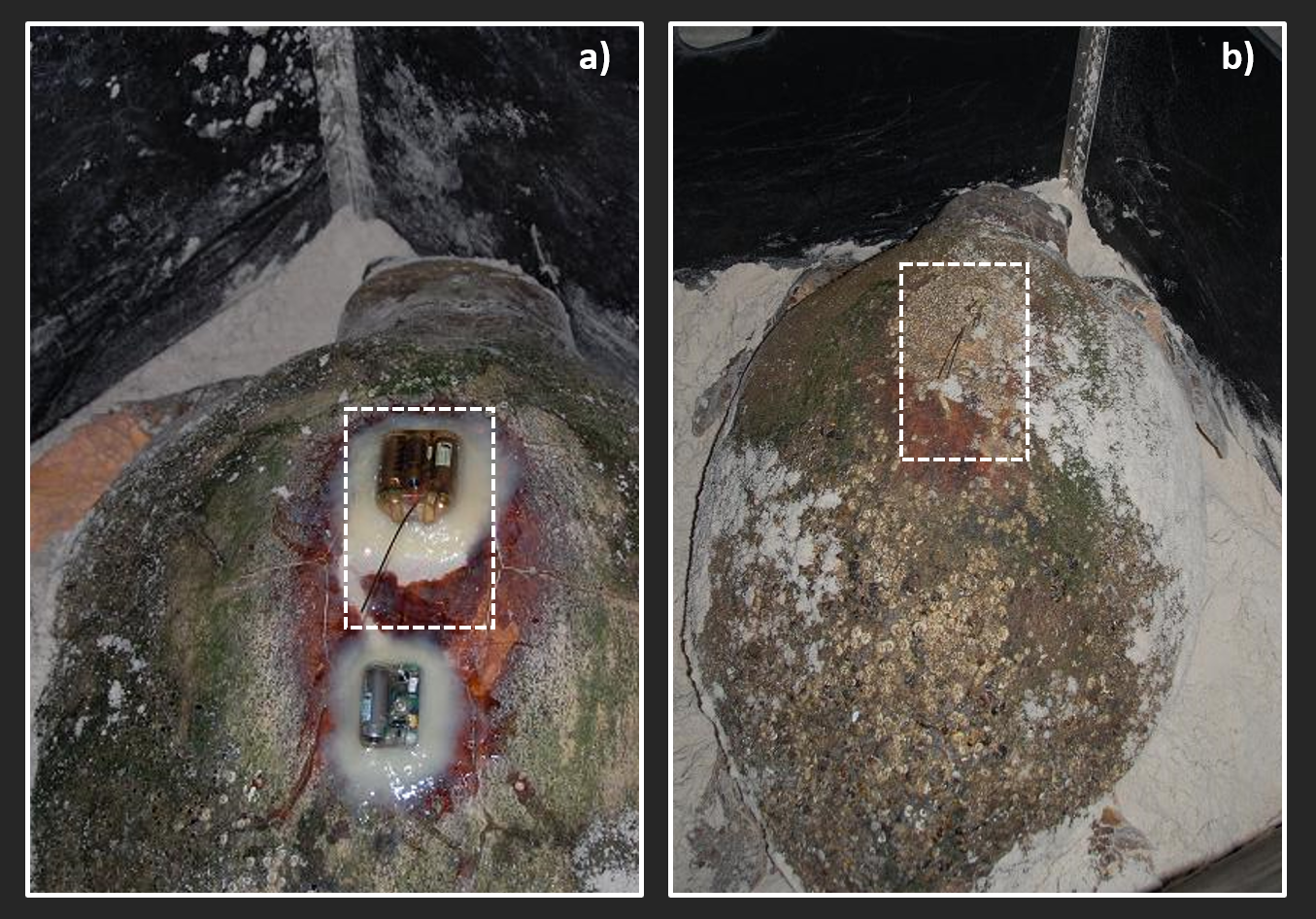

Supplement: Supplementary file 3 — Additional file 3. Example of SPOT tag fouling by marine organisms on a loggerhead turtle (Caretta caretta) in the northern Gulf of Mexico. Tag was attached a) June 13th and b) 25 days later (July 8th, 2013) the tag is covered primarily with barnacles, although the antenna is visible. Photograph by the U.S. Geological Survey. [file 40462_2020_237_MOESM3_ESM.docx]
