# Supplementary material for: Drivers of realized satellite tracking duration in marine turtles
Source: Mov Ecol. 2021 Jan 5;9:1. doi: 10.1186/s40462-020-00237-3 (PMC7786511; doi:10.1186/s40462-020-00237-3)

Additional file 4. Methodology used to assign marine turtles to foraging regions by plotting satellite tag location data (panels a, c, e) and plotting cumulative distance traveled over time per individual (panels b, d, f). Visualizing these data can indicate the point in time where distance traveled begins to level out/reach an asymptote (i.e., red dashed lines). We designated foraging sites near the asymptote. Examples include a) a nesting female departing the Dry Tortugas and arriving at a foraging site in the Bahamas, c) an in-water captured male resident of the Dry Tortugas, who made some looping movements away from, and then back to, the Dry Tortugas, and e) a male migrating from the waters offshore of Cancun, Mexico presumably to breed, then exhibiting return migration to the foraging site at the Dry Tortugas. Grey shaded boxes in panels b and f indicate migration intervals; no migration is occurring in panel d for this resident turtle.


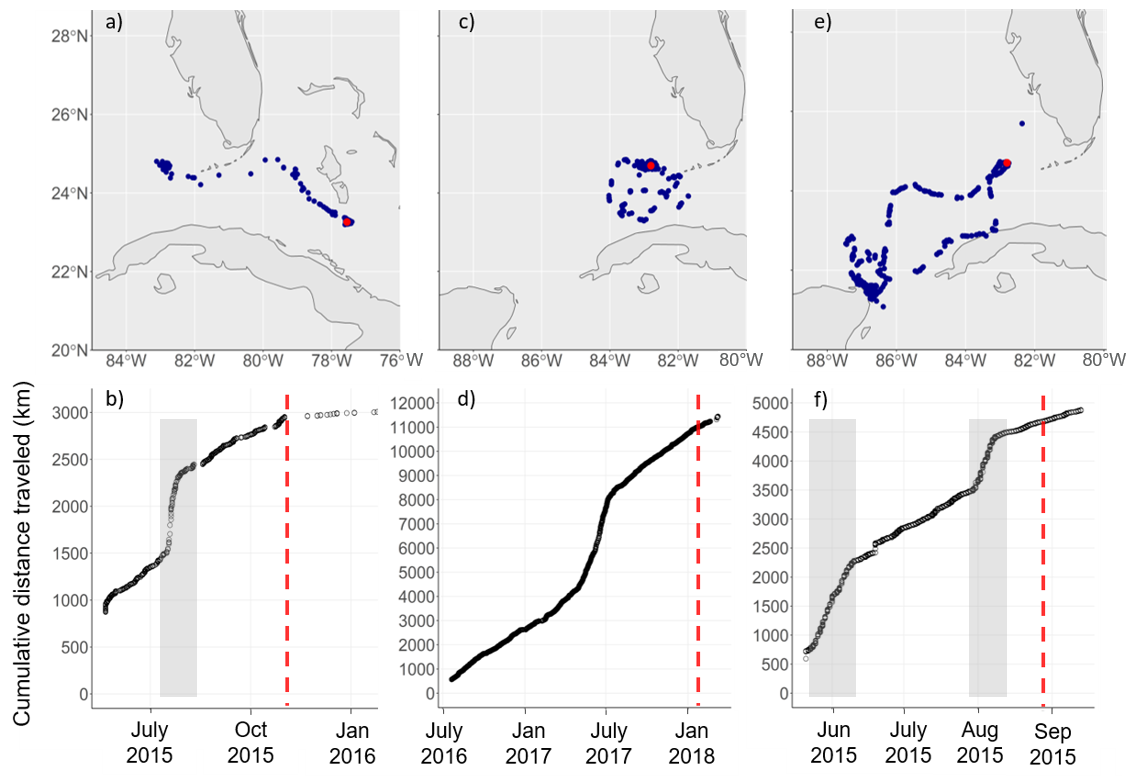

Supplement: Supplementary file 4 — Additional file 4. Methodology used to assign marine turtles to foraging regions by plotting satellite tag location data (panels a, c, e) and plotting cumulative distance traveled over time for each individual (panels b, d, f). Visualizing these data can indicate the point in time where distance traveled begins to level out/reach an asymptote (i.e., red dashed lines). We designated foraging sites near the asymptote. Examples include a) a nesting female departing the Dry Tortugas and arriving at a foraging site in the Bahamas, c) an in-water captured male resident of the Dry Tortugas, who made some looping movements away from, and then back to, the Dry Tortugas, and e) a male migrating from the waters offshore of Cancun, Mexico presumably to breed, then exhibiting return migration to the foraging site at the Dry Tortugas. Grey shaded boxes in panels b and f indicate migration intervals; no migration is occurring in panel d for this resident turtle. [file 40462_2020_237_MOESM4_ESM.docx]
